# Testing the Dispersion of Nanoparticles in a Nanocomposite with an Ultra-Low Fill Content Using a Novel Non-Destructive Evaluation Technique

**DOI:** 10.3390/ma15031208

**Published:** 2022-02-05

**Authors:** Nicola Montinaro, Mario Fustaino, Denise Bellisario, Fabrizio Quadrini, Loredana Santo, Antonio Pantano

**Affiliations:** 1 Department of Engineering, University of Palermo, 90128 Palermo, Italy; nicola.montinaro@unipa.it (N.M.); mario.fustaino@unipa.it (M.F.); 2 Département D’astronomie, Faculté des Sciences, Université de Genève, 1211 Geneve, Switzerland; 3 Faculty of Economics, Universitas Mercatorum, 00186 Rome, Italy; denise.bellisario@unimercatorum.it; 4 Department of Industrial Engineering, University of Rome “Tor Vergata”, 00133 Rome, Italy; fabrizio.quadrini@uniroma2.it (F.Q.); loredana.santo@uniroma2.it (L.S.)

**Keywords:** nanocomposite, NDE, thermographic inspection, IR-NDT, PPT, metal nano-particles, nano-coating, additives

## Abstract

A non-destructive evaluation (NDE) technique capable of testing the dispersion of nanoparticles in a nanocomposite would be of great use to the industry to check the quality of the products made and to ensure compliance with their specifications. Very few NDE techniques found in the literature can evaluate the level of dispersion of the nanoparticles in the whole nanocomposite. Here, a recently developed NDE technique based on pulsed phase thermography (PPT) in transmission mode was used to assess the particle dispersion in ultra-low, less than 0.05 wt%, Ag enriched polymeric based nanocomposite manufactured with an innovative nano-coating fragmentation technique. The phasegrams obtained with the presented technique clearly showed clusters or bundles of Ag nanoparticles when present, down to the size of 6 µm. Therefore, the new NDE approach can be applied to verify that the expected levels of dispersion are met in the production process.

## 1. Introduction

In recent years, nanocomposites (NCs) are gaining more and more attention from the industry [1]. NCs found application in various fields, such as material manufacturing [2], engineering [3], transport [4], electronics [5], food and beverages [6,7], aerospace [8] and pharmaceuticals [9], thanks to their improved properties, with particular regards to mechanical properties [10,11], thermal conductivity in polymers with SWCNT [12,13] and with MWCNT [14], electrical conductivity in NCs with MWCNT [15,16], as well as in the case of AC current with different frequency values [17], graphitized CNT [18], and optical properties in NCs with SWCNT [19,20] and MWCNT [21,22]. The properties of the resulting nanocomposite are strongly dependent on the type of nanoparticles added to the matrix [23,24]. When nanocomposite materials with anti-bacterial properties are required, silver nanoparticles, thanks to their good biocompatibility and anti-bacterial affinity, can be used [25]. In [26], the recent fabrication technique by fragmentation is explained, while [27] provides a historical review of the use of silver in this field; in [28], silver compounds were used.

It is well known that the level of dispersion of the nanoparticles in the polymer matrix is the parameter that, much more than others, can influence their enhancement capabilities [29,30,31]: a good dispersion level increases the surface contact between the nanoparticles and the matrix and, thus, facilitates their interactions.

Although an extensive use of nanocomposites is desirable, both their production and the testing of the finished products are rather difficult. It is necessary to obtain nanoparticles of the desired size and shape and to avoid their aggregation during the manufacturing phase. Furthermore, it is essential to have low-cost non-destructive evaluations (NDE) to control the quality of the products made, in order to guarantee compliance with their specifications.

Very few NDE techniques able to evaluate the level of dispersion of the nanoparticles in the whole NCs can be found in the literature. One is based on the impulse acoustic microscopy method [32], but this NDE method is slow and can scan only small samples. Dynamic scanning calorimetry (DSC) was proposed in [33] to test the dispersion of carbon nanotubes (CNTs). According to the authors of [33], the exothermic reaction heat during the curing process could allow one to estimate the degree of CNT dispersion in NCs; however, this approach cannot be used on the final composite and, therefore, seems to be of limited utility.

Promising novel non-destructive techniques, capable of testing the dispersion of nanoparticles in NCs, rely on infrared thermography [34,35,36]. In [34], nanocomposites samples with different levels of nanoparticle dispersion were manufactured and tested by step heating thermography (SHT), showing differences in the thermal response over the time transient. The limitation of this method is that it works only as a comparison between pairs of specimens placed side by side. The pulsed phase thermography (PPT) approach [35,36] allows one to overcome this limitation, making it possible to detect the aggregates, thus, discriminating between well dispersed and poorly dispersed samples. The PPT approach consists in the pulsed thermography (PT) technique with the phase study, as described by Maldague et al. [37,38], and uses thermal images [39,40] to combine the advantages of PT and lock-in thermography. In the PT, a sample surface is pulse heated by a flash lamp, generating a series of thermal waves (with varying amplitudes and frequencies) that propagates through the thickness of the material. The full field superficial temperature decay (after the pulse) is then monitored by an infrared camera and by the phase content retrieved with the aim of the discrete Fourier transform algorithm [35,37]. The harmonic content of the acquisitions, shown in the form of phasegrams, could indicate the local phase contrast due to the thermal inhomogeneity and, thus, the clusters of nanoparticles.

In the PPT technique, it is expected that deeper clusters are better contrasted at low frequency phasegrams, where shallow aggregates are at higher frequency phasegrams. The signal normalization inherent in the evaluation of the phase also serves to reduce the counter effects of the non-uniform heat deposition and/or environment reflections [36,40].

The easy implementation and the ability to scan large surfaces have quickly rendered the PT setup widely used in the aerospace field for the damage assessment of composite parts [41,42]. In [43], the phasegrams generated with the modulated frequency thermography and the PPT approach are compared on aerospace grade composite to characterize bonding defects. In order to get the best results in the PPT technique, the sample rate of the IR-camera must accord with the thermal diffusivity of the inspected material. Higher thermal diffusivity material requires a higher sampling rate to fit with the cooling transient speed.

In [44], the PPT technique was successfully applied to evaluate the dispersion level in CNT-based nanocomposites. Montinaro et al. used samples with different dispersion levels of the nanoparticles as a benchmark for the proposed approach, which allowed the discrimination of the CNTs aggregate up to a resolution of 0.18 mm [44].

In this work, an alternative PPT approach was developed in order to enhance the detecting resolution in respect to the work by Montinaro et al. [44]. The alternative method performances were evaluated in assessing the particles dispersion in ultra-low (less than 0.05 wt%) enriched polymeric-based nanocomposite manufactured with a nano-coating fragmentation technique described in [45], based on an injection molding process. The outcomes of the technique can be used to optimize the production (tuning the manufacturing parameters of the process) to enhance the dispersion and/or to test the quality of the manufactured polymer nanocomposites.

It is worth highlighting further the novelty of this study compared to the previous ones. The technique presented in 2018, ref. [34], is rather different; there, we used step heating thermography (SHT) to show differences in the thermal response over the time transient, while, here, we used pulsed phase thermography (PPT). Moreover, even if, in the 2020 publication, ref. [44], we used the PPT, there are several differences with the presented paper; here, there is a significant enhancement of the energetic and spatial resolution (from 0.18 mm/px to 6 µm/px) performed with the aim of a magnification lens and the adoption of an alternative experimental setup in transmission mode, which, in ref. [44], was in reflection mode. The PPT data analysis for the transmission mode setup have led to totally different considerations.

## 2. Materials and Methods

### 2.1. Nanocomposite Manufacturing

Nanocomposites were manufactured by means of the innovative nano-coating fragmentation (NCF) technique. Thermoplastic pellets for injection molding are coated with thin continuous layers of the filler. In the plastification stage, the continuous layer is fragmented in small platelets that are distributed into the resin matrix. If a technology for nanometric films is used for pellet coating such as physical vapor deposition (PVD), nano-platelets will result in the molded nanocomposite. This manufacturing procedure enters the class of the melt mixing processes for nanocomposite production but solves many problems related to the use of free nanoparticles. In fact, in conventional melt mixing procedures, clusters of nanoparticles are difficult to disaggregate, thus, affecting properties of final products. Higher contents are necessary to allow minimal distribution of nanoparticles into the matrix, and full exfoliation is quite impossible due to the high viscosity of thermoplastic resins. Moreover, the handling of nanoparticles is an issue for the safety of workers and for the environment. In NCF, nano-platelets are produced during, and not before, the plastification stage. Large clusters and agglomerates are absent. As a result, very low contents may be obtained with optimal distribution into the matrix.

In the current experimentation, silver-polypropylene (Ag/PP) nanocomposites were injection molded into 80 × 80 × 3 mm^3^ plates. A commercial PP (Moplen HC500N, LyondellBasell Industries Holdings, Brindisi, Italy) was used in the shape of pellets with 0.9 g/cm^3^ density and size in the range 2.5–4.8 mm. The silver filler was made from a PVD target (99.99% of purity) with a large rectangular shape (300 × 125 mm^2^).

**Figure 1:**
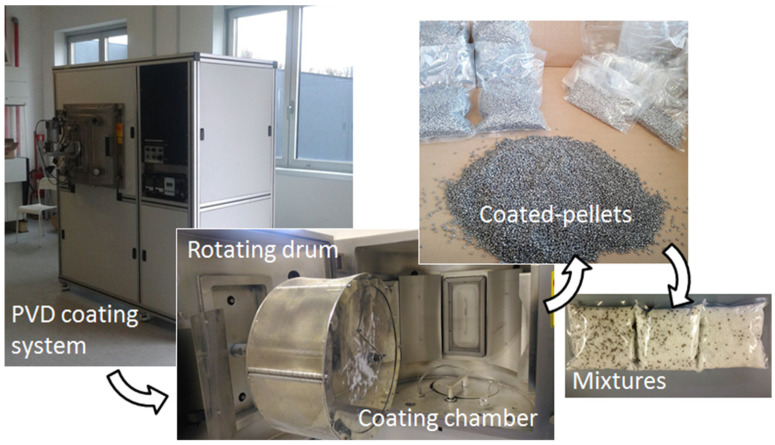
PVD coating systems and coated pellets.

The system for PVD deposition (by MITEC srl, Rome, Italy) is shown in Figure 1 together with coated pellets and their mixtures. This machine has a large vacuum chamber (about 600 mm in maximum size) where a rotating drum is placed for pellet coating. This machine architecture has been developed to allow for coating large amounts of pellets for a single batch. The pellets are inserted into the drum which rotates around an axis parallel to the ground. A net in the external surface of the drum allows the passage of Ag nanoparticles, which are produced during the PVD process. Coatings were made with batches of 1 kg of PP pellets, at the DC power of 400 W, a drum rotational speed of 29 rpm, and a deposition time of 20 min under an argon gas flux (purity of 99.999%) of 1.00 L/min during sputtering.

Thickness of pellet coating was evaluated by Ag extraction in 1 M nitric acid. In fact, nitric acid completely dissolved the metal coating without affecting the PP substrate. A small amount (about 10 g) of coated pellets were dried and immersed into the acid. The solution was stirred at the temperature of 120 °C for 2 h. Weight measurements were carried out before and after Ag removal. A silver content of roughly 0.05 wt% was estimated on the average of three measurements. By assuming a spherical shape and an average diameter of 2 mm for the PP pellets, an average thickness of about 60 nm was inferred. Moreover, considering that all the silver of the coatings moved into the final composites, the same final content of 0.05 wt% was expected for them.

**Figure 2:**
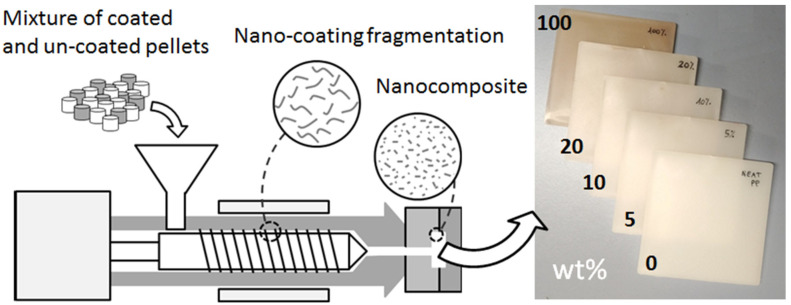
Injection molding process for the manufacturing of the nanocomposites.

The concept of NCF by injection molding is depicted in Figure 2. Mixtures of coated pellets with virgin pellets were prepared with percentages ranging from 0 (neat PP) to 100% of deposited pellets. Five compositions were chosen (0, 5, 10, 20, and 100% of coated pellets), which corresponded to five expected Ag contents in the molded nanocomposites (0, 0.0025, 0.05, 0.01, and 0.05 wt%, respectively). A full electric press (Roboshot S-2000i 50B by FANUC Italia, Milan, Italy) was used for manufacturing. The molding parameters were selected according to the practice of PP processing (45 mm/s injection speed, 1200 bar maximum pressure, 5 s injection time, 15 s cooling time, and 280 °C nozzle temperature).

From here on, the sample type will be labeled as follows: neat, 5%, 10%, 20% and 100%, where the percentage indicated is relative to the quantity of coated pellets added and, therefore, they corresponded to the following percentage of weight fraction, %wt, of Ag particles in the nanocomposite: 0, 0.0025, 0.005, 0.01, 0.05.

**Figure 3:**
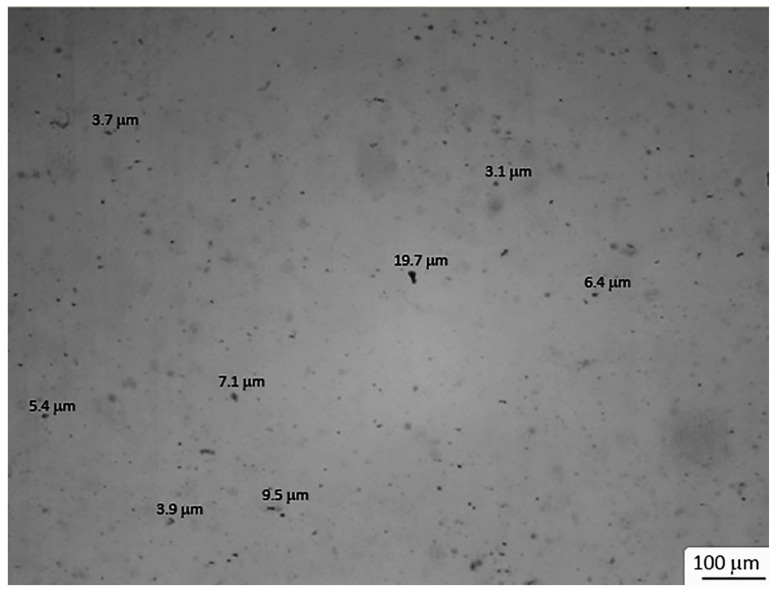
Micrographs of the nanocomposite prepared with 100% of coated pellets, which correspond to a 0.05 wt% of Ag nanoparticles.

In Figure 3, an optical evaluation of one sample of nanocomposite was obtained via micrographs. The sample manufactured with 100% of coated pellets, which corresponded to a 0.05 wt% of Ag nanoparticles, was selected in order to maximize the possibility of detecting the presence of bundles of particles.

### 2.2. Pulsed Phase Thermography Setup

In the classic PT setup, an IR-camera and heat source are placed on the same side in respect to the sample in a configuration called reflection mode. This setup is generally preferred because it is easier to deploy given that both sides of the specimen are not always available. Moreover, the reflection setup gives the best results when the defects to be inspected are closer to the heated surface. If both surfaces of the sample are accessible, an alternative viable setup for the PT is the transmission mode, where the IR-camera and the heat source are placed on opposite sides of the sample.

In the transmission mode, the travel distance of the thermal wave is fixed and equal to the sample thickness *t*, while, in reflection mode, this depends on the position *d* of the anomaly (the reflector) in respect to the pulsed surface; this implies that, for deeper inspections (*d* > *t*/2), the transmission setup is preferred, showing fewer attenuated signals. On the other hand, depth information is lost in the transmission setup because thermal waves will travel the same distance and, hence, depth quantification will not be possible anymore [38]. Another advantage of the transmission setup is the possibility to enhance the spatial resolutions of the IR-camera, reducing the focal distance by adopting spacer rings installed on the lens. The adoption of a focal length of a few centimeters would definitely enhance the resolutions (with the same equipment) but would neglect the positioning of the heat source on the same side of the IR-camera (the reflection setup), because a uniform irradiation would no longer be possible. In conclusion, if the main aim of the NDE is to foster the defect sensitivity eluding the depth quantification, the transmission mode can be considered as a valuable alternative.

**Figure 4:**
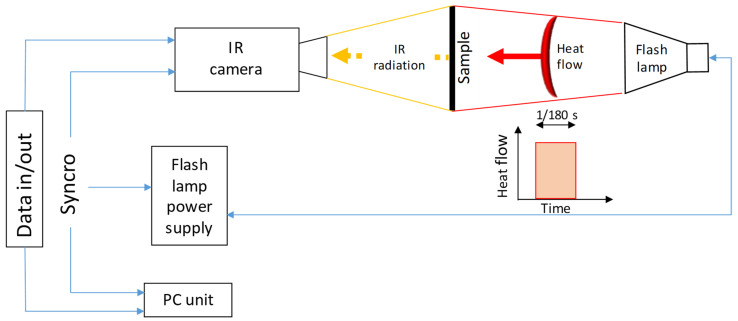
Schematic representation of the PPT experimental in transmission setup.

In order to assess the dispersion of particles on samples that are not excessively thick, the benefit of the transmission PT experimental setup arose with better thermal contrast and the possibility to enhance the spatial resolution with a short focal length. In Figure 4, the schematic representation of the adopted PPT transmission experimental setup is shown.

As a heat source, an Elinchrom flash lamp that releases a pulse of 4800 W/s powered by two Elinchrom 2400 rx in parallel configuration was used. The surface temperature cooling transient was acquired with an InSb cooled IR-camera (FLIR Systems, Wilsonville, OR, USA) FLIR X6540sc that was equipped with a 50 mm F/2.0 lens (field of view H × V = 21.74° × 17.46°) and a spacer ring to increase the spatial resolution (thus reducing the focal length to 4 cm). With this combination, the obtained resolution was 6 µm/px. The set value of the integration time is a trade-off between energetic resolution, sample rate and the need to not saturate the IR detector. It is well-known that higher integration time enhances the energetic resolution but decreases the sample rate and facilitates the saturation of thermograms.

In Table 1, a summary of the setup parameters used for the experiment is reported.

The IR-camera acquisition was triggered by the single pulse of the flash lamp and stopped with an internal clock of the IR-camera software once the temperature gradient became negligible.

Since a transmission setup was used, special attention has been paid to avoid the possibility that the heat pulse radiation could arrive directly on the lens of the camera, saturating the thermogram; thus, any gaps between sample and sample-holder were filled.

## 3. Results

**Figure 5:**
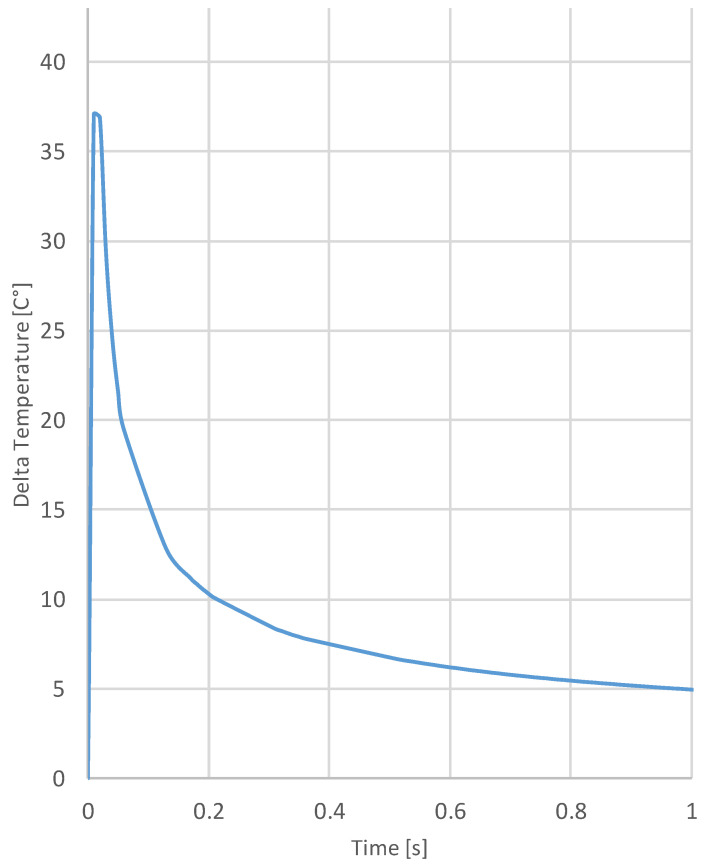
Delta temperature vs. time trend of the central area of one sample.

In the PT setup, the single heat pulse generated a series of thermal waves into the sample at different frequencies that normally propagate to the injection surfaces. As discussed before, the thermal waves are then captured in the form of IR-radiation by the IR-camera placed on the opposite side of the sample. The arrival of the thermal wave propagating along the cross-section of the sample to the opposite surface corresponds to the peak shown on the graph of Figure 5, where the mean delta temperature vs. the time is extracted from a central area of the time-series of thermograms for one of the samples.

If the thermograms acquired in transmission mode are analyzed in the time domain, the wave passing through the anomalies with lower thermal conductivity than the surrounding environment (the matrix) will be detected as an attenuated area in the thermogram. Conversely, in the case of higher thermal conductivity in respect to the environment, the wave passing through the anomalies will be detected as less attenuated areas on the thermogram. In both cases, a thermal contrast should be noted.

Since the aim of this work is to detect agglomerates of particles to assess the quality of their dispersions, the weak thermal contrast given by such small discontinuity could be difficult to observe. For this reason, the data presentation in the function of time is not considered as a valid solution. Analyzing the cooling transient (after the peak of Figure 5) in the frequency domain, by the application of the discrete Fourier transform (DFT), the phase of the harmonic content is retrieved from each pixel. The results can be shown in graphical form as a phasegram image mitigating the effect of environmental reflections, emissivity variations, and non-uniform heating, as well as surface geometry and orientation enhancing contrast changes and, thus, detectability.

It is worth noting that, in the application of the PPT algorithm on a reflection mode acquisition, phasegrams at a lower frequency correspond to a deeper probing distance from the surface and, conversely, at a higher frequency, to shallower inspection; however, this is not the case for the transmission mode, since the thermal wave will not be reflected by the discontinuity [36].

**Figure 6:**
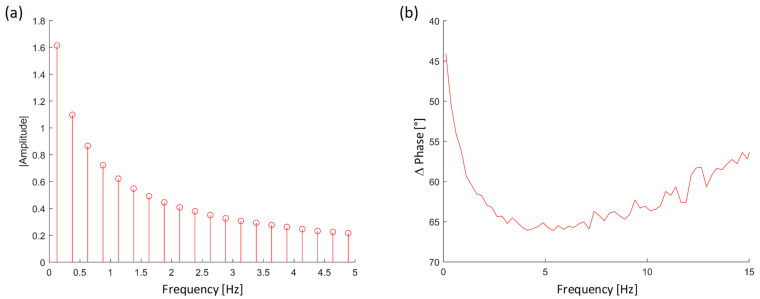
Stem graphs reporting the absolute value (**a**) and the phase of the Fourier Transform (**b**) of a generic pixel.

In Figure 6a, stem graphs report the absolute value of the Fourier transform, corresponding to the average heat power injected by each harmonic. The graphs are obtained from the time response on a generic point lying on the sample surface. The phase content of the same pixel is calculated as a function of the frequency in Figure 6b.

Looking at the plot of Figure 6a, it is possible to note that the main harmonic contribution is found at thermal wave frequencies up to 5 Hz.

**Figure 7:**
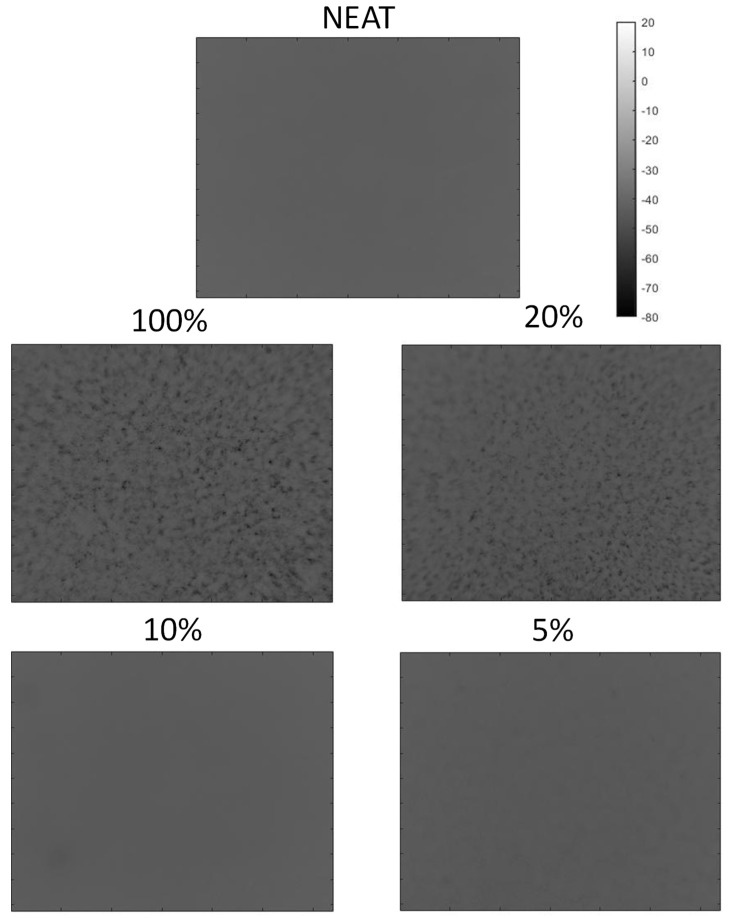
Phasegrams calculated at 0.12 Hz (low frequency) for the five tested samples with different fraction of nanoparticle content.

In Figure 7, the phasegrams retrieved with the application of the PPT algorithm at the lowest frequency of 0.12 Hz are presented. Looking at the phasegrams sequence, starting with the Neat sample, a clear trend between particle percentage and amounts of aggregates was found.

In particular, in the Neat, 5% and 10% samples, the reported phasegrams were both uniform. As defined in the Section 2.1, the indicated percentage were relative to the number of coated pellets of the total, meaning that, for the 100% sample, the weight fraction of Ag particles was 0.05%. In 20% and 100% samples, with a higher fraction of particles, some inhomogeneity, represented as clusters with different thermal responses, could be detected, with the 100% sample standing out for amounts and size.

**Figure 8:**
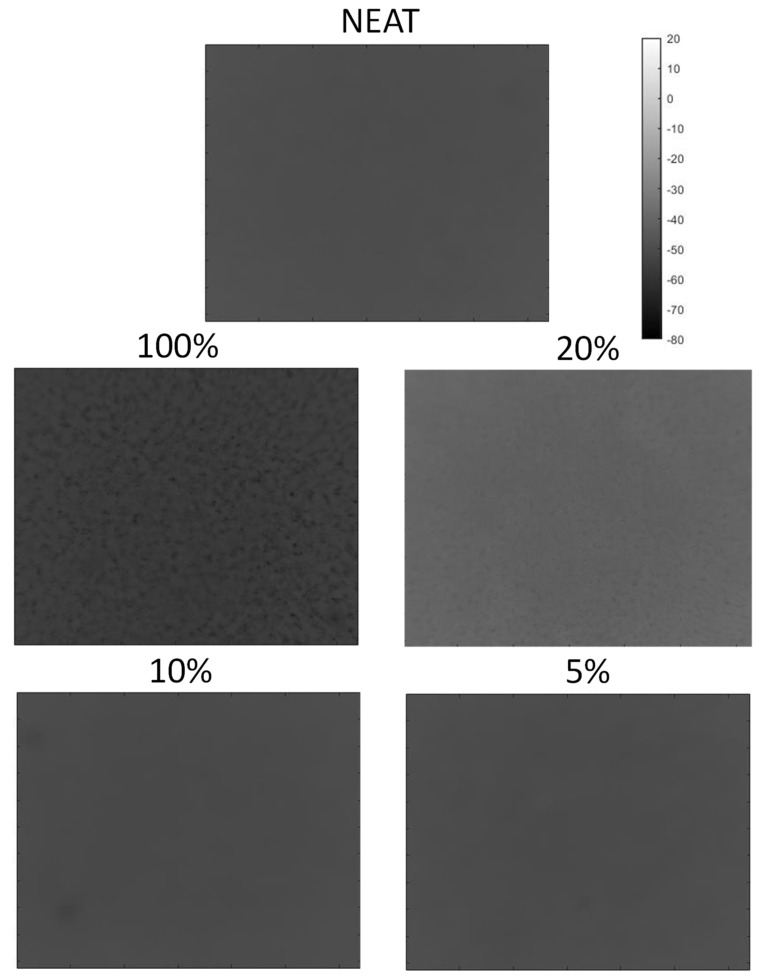
Phasegrams image for the five samples calculated at 0.37 Hz (high frequency).

In Figure 8, the phasegrams for the five samples at different filler percentages are extracted at the higher frequency of 0.37 Hz to confirm the previous results.

Here, the samples without added particle and with low percentages (Neat, 5% and 10%) reported an even phase, while the 20% and the 100% samples confirmed the outcome of the previous analysis at a lower frequency. For the 20% sample, less phase contrast in respect to the lowest frequency (0.12 Hz) was shown. Comparing the analyses performed at the low and high frequency, the presence of aggregates for the 20% and 100% samples was consistent with same shape and location if they overlapped with a slightly lower contrast for the higher frequency. Increasing the frequency of the phasegram from 0.12 Hz to 0.37 Hz did not lead to better results, confirming that only lower frequency must be selected for this purpose.

**Figure 9:**
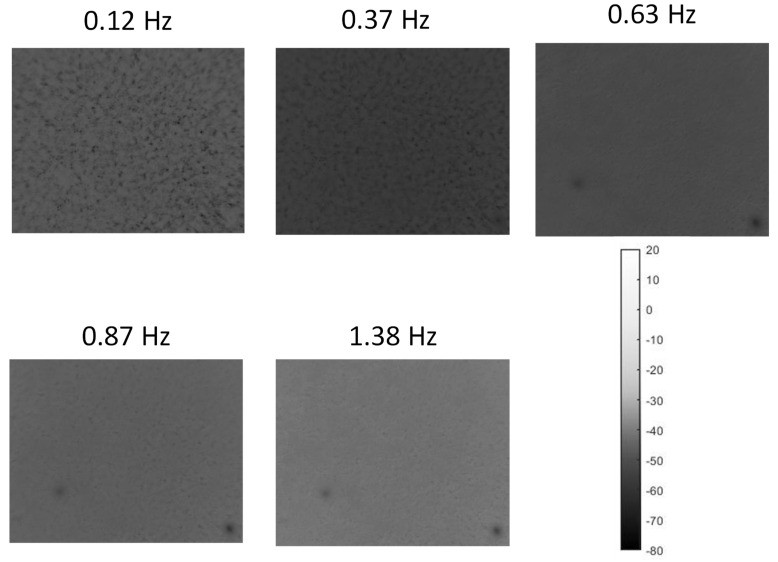
Sequence of phasegrams at gradually increased frequencies calculated for the 100% sample.

In Figure 9, a sequence of phasegrams at a gradually increased frequency for the 100% sample is reported. As expected, increasing the selected frequency gradually reduced the phase contrast of the discontinuity and, thus, the detecting capability of the technique.

**Figure 10:**
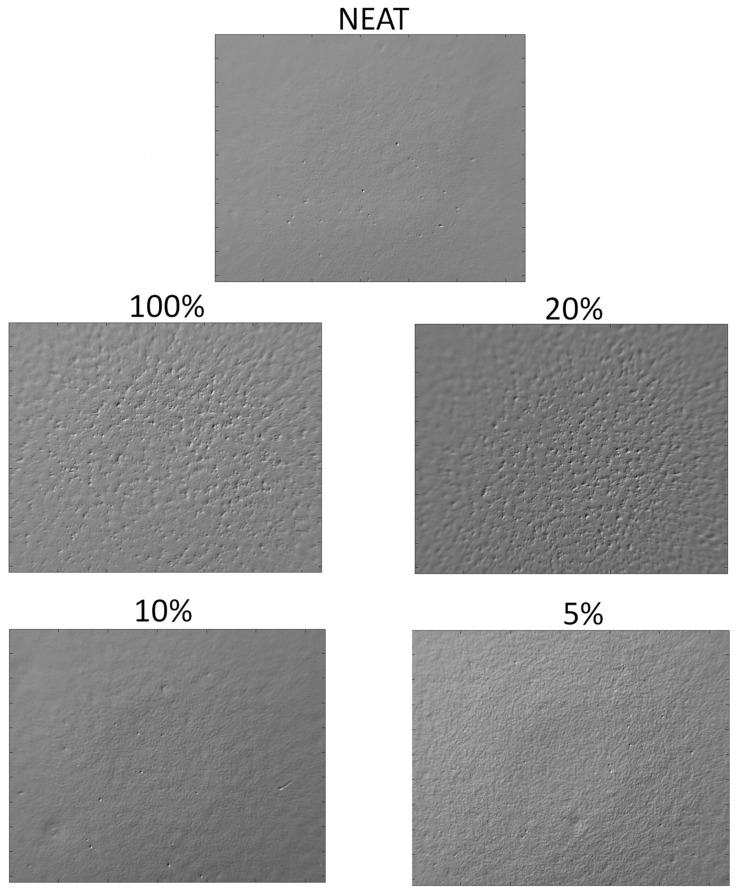
Gradient visualization calculated in two-dimensional functions.

A data presentation strategy able to emphasize the phase contrast and, thus, visualize the discontinuities inside the samples, is the two-dimensional gradient function that is reported in Figure 10 for the five samples.

The gradient visualization is useful for calculating the sum of the derivatives in the planar x-y directions. The presentation allows one to highlight the variability of a quantity in an x-y position and is useful in images to distinguish when passing from a discontinuity to a uniform zone since the quantity that is taken into consideration is the value assumed by the various pixels, which, in this case, is the phase. If the gradient is high, it means that there is a strong variation in the value assigned to the adjacent pixels in the image and, therefore, that it is highly probable that, in that area, there is a discontinuity.

## 4. Discussion

The cluster detection strategy presented in this work is based on the assumption that a thermal wave propagating through the sample thickness, once having passed a discontinuity with a different thermal conductivity, is shifted in phase due to the involved complex scattering process, which is not the topic of this study. The phase shift can be presented in the form of an image by applying the DFT on the acquired thermogram and selecting the desired frequency.

The high spatial resolution required to detect small discontinuity was obtained by reducing the focal length of the IR-camera (up to few centimeters from the sample); thus, the transmission mode setup was adopted instead of the reflection one, allowing for a fair heat injection from the free sample surface.

Even if the scanned area of the sample is confined (5 mm × 3 mm), it can be considered as a region of interest that, since it is equal for all the samples, offers a clear indication of the quality of the dispersion and of the aggregation of the particles in a batch. Furthermore, the acquisition procedure takes less than a second for a single shot; thus, with the aim of a robotized positioner, larger areas could be quickly inspected if required.

The indications extracted from the phasegrams obtained in the transmission configuration are more likely to be representative of the sample surface closest to the IR camera than that of the heat source, since the attenuation and diffusion processes are taking over with distances. Since the samples tested here are quite thin, further acquisitions by flipping the sample (reversing the positions of the IR camera with the heat source) were not necessary. Clearly, the transmission setup does not provide any information on the depth of the detected anomalies because all the outputs are flattened on a single plane.

Consequently, this technique is useful and easy to implement in detecting clusters and agglomerates of particles with thermal conductivity that is different than the matrix for the evaluation of the quality of dispersion within products coming from manufacturing processes. Indeed, from the outcomes shown in Figure 7, a clear difference between samples with low percentage enrichments and the others can be noted, suggesting that, for samples with a higher percentage, the manufacturing process can still be improved. The latter statement does not mean that, in the samples here declared with even dispersion, there are not agglomerates, but rather that they are too small to be detected.

As shown by the frequency analysis in Figure 9, phasegram selection is crucial to improve thermal contrast. In particular, with the current samples’ geometry and the material’s thermal specifications, the lower frequency showed better contrast. A preliminary analysis is always suggested to optimize the results.

An alternative image presentation that uses the gradient of the phase-shift is reported in Figure 10, offering a peculiar tridimensional deep contrasted image representative of the detected clusters.

The micrographs shown in Figure 3 confirm the presence of a large amount of agglomerates larger than 3 µm. Taking into consideration that the PPT technique is used in transmission mode, where the IR camera and the heat source are positioned on opposite sides of the sample and that, therefore, all the clusters along the thickness are detected, and that an acquisition of a thermocamera provides boundaries that are blurred with respect to an optical image, since it measures temperature variations between different areas, the results of the NDE technique seemed to agree with the optical evaluation.

## 5. Conclusions

The dispersion quality of the nanoparticles in the polymer matrix is the parameter that, much more than others, affects the performances of the final manufact. A good dispersion level guarantees the surface contact between the nanoparticles and the matrix and, thus, between their interactions. In a previous work, five types of polypropylene (PP) nanocomposites samples with different levels of Ag nanoparticles infill have been manufactured with a new technique. The method first consisted in a metal nano-film deposition on PP pellet substrates by physical vapor deposition (PVD) sputtering equipment. Subsequently, a percentage of the coated pellet was mixed to uncoated ones via an injection molding machine to produce the polymer nanocomposites in a single step. In order to assess the dispersion of particles on samples that were not excessively thick, the benefit of the transmission PT experimental setup showed better thermal contrast and the possibility to enhance the spatial resolution with short focal length.

In this work, the pulsed thermography in transmission mode was applied on five nanocomposite samples for testing the dispersion of the added filler. The NDE technique is based on a pulsed phase thermography (PPT) approach, wherein the acquisition procedure of the “pulsed thermography” (PT) is combined with the phase/frequency concepts of “lock-in thermography” for which specimens are submitted to a periodical excitation. The proposed infrared-NDT techniques allow for fast non-contact inspections of the nanocomposite with a flexible hardware setup. The phasegrams obtained with the presented technique clearly show clusters or bundles of Ag nanoparticles when present, down to the size of 6 µm. Thus, we can state that the new technique proved to be useful and effective for the individuation and the study of the inner structure of nanocomposites with different kinds of filling materials.

## Figures and Tables

**Table 1:** Specifications of the parameters used for the PPT setup.

PPT Parameter Setup	Value
Distance sample to IR-camera	40 ± 2 mm
Distance flash lamp to sample surface	130 ± 2 mm
Sample rate IR-camera	100 Hz
Integration time	2000 µs
Heat flow duration	1/180 s

## Data Availability

Data available on request due to restrictions.
